# Real‐Time and Site‐Specific Perturbation of Dynamic Subcellular Compartments Using Femtosecond Pulses

**DOI:** 10.1002/smsc.202500166

**Published:** 2025-05-22

**Authors:** Seohee Ma, Bin Dong, Matthew G. Clark, R. Michael Everly, Shivam Mahapatra, Chi Zhang

**Affiliations:** ^1^ James Tarpo Jr. and Margaret Tarpo Department of Chemistry Purdue University 560 Oval Dr. West Lafayette 47907 IN USA; ^2^ Purdue Institute for Cancer Research Purdue University 201 S. University St. West Lafayette 47907 IN USA; ^3^ Purdue Institute of Inflammation, Immunology, and Infectious Disease Purdue University 207 S. Martin Jischke Dr. West Lafayette 47907 IN USA

**Keywords:** femtosecond laser, low‐density plasma, microsurgery, mitochondria, pulse‐picking, reactive oxygen species, real‐time precision opto‐control

## Abstract

Understanding laser interactions with subcellular compartments is crucial for advancing optical microscopy, phototherapy, and optogenetics. While continuous‐wave lasers rely on linear absorption, femtosecond (fs) lasers enable nonlinear multiphoton absorption confined to the laser focus, offering high axial precision. However, current fs laser delivery methods lack the ability to target dynamic molecular entities and automate target selection, making them incapable of performing real‐time perturbation of mobile or complexly distributed biomolecules. Additionally, existing technologies separate fs pulse delivery and imaging, preventing simultaneous recording of cellular responses. To overcome these challenges, this study introduces fs real‐time precision opto‐control (fs‐RPOC), which integrates a laser scanning microscope with a closed‐loop feedback mechanism for automated, chemically selective subcellular perturbation. Fs‐RPOC achieves superior spatial precision and fast response time, enabling single‐ and sub‐organelle microsurgery of dynamic targets and localized molecular modulation. By applying a pulse‐picking method, fs‐RPOC independently controls laser average and peak power at any desired subcellular compartment. Targeting mitochondria, fs‐RPOC reveals site‐specific molecular responses resulting from fs‐laser‐induced reactive oxygen species formation, H_2_O_2_ diffusion, and low‐density plasma generation. These findings offer new insights into fs laser interactions with subcellular compartments and demonstrate fs‐RPOC's potential for precise molecular and organelle regulation.

## Graphical abstract

This work introduces femtosecond real‐time precision opto‐control (fs‐RPOC), a technology enabling automated, chemically selective subcellular perturbation with precisely controlled laser dosage confined to designated targets. Fs‐RPOC achieves high spatial precision suitable for 3D sub‐organelle microsurgery. Furthermore, it facilitates the investigation of cellular responses to localized perturbations induced by reactive oxygen species, and low‐density plasma.

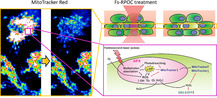

## 1 Introduction

Understanding how cells and subcellular compartments respond to light interactions is essential for advancing optical microscopy, phototherapy, and optogenetics. Light‐matter interaction mechanisms in biological systems vary with wavelength. Ultraviolet (UV) light can generate reactive oxygen species (ROS) or directly damage DNA,^[^ ^1^ ^]^ while mid‐infrared photons primarily cause photothermal effects by matching the photon energy with the vibrational absorption energy of biomolecules.^[^ ^2^ ^]^ The near‐infrared (NIR) region is particularly advantageous for biological imaging due to reduced photon absorption and minimal off‐target effects. Pulsed lasers, particularly femtosecond (fs) lasers, interact with biomolecules in ways distinct from continuous‐wave (CW) lasers. While biomolecules exhibit relatively weak linear absorption of NIR fs pulses, the high peak power of these laser pulses facilitates multiphoton absorption, a nonlinear process that can excite fluorescent molecules, generate ROS, induce localized heating, and activate photoresponsive proteins exclusively at the laser focus.^[^ ^3^, ^4^ ^]^ At higher peak power, fs lasers can lead to multiphoton ionization and low‐density plasma (LDP) generation, resulting in more pronounced perturbations to local molecules.^[^ ^3^, ^5^ ^]^

Low‐energy fs lasers are widely used in biological imaging modalities, such as multiphoton‐excitation fluorescence (MPEF),^[^ ^6^ ^]^ transient absorption,^[^ ^7^ ^]^ and stimulated Raman scattering (SRS) microscopy.^[^ ^8^ ^]^ NIR fs pulses enable deeper tissue penetration with minimal impact on objects outside the focal plane.^[^ ^6^ ^]^ Nonlinear optical effects further ensure that optical signals from multiphoton processes are restricted to the focal plane, providing intrinsic optical sectioning capability. Beyond imaging, fs pulses have been used to induce localized perturbations to biological samples. For example, fs laser pulses targeted to the endoplasmic reticulum have been shown to trigger calcium leakage into the cytosol.^[^ ^9^, ^10^, ^11^, ^12^ ^]^ Fs lasers have also been employed for nanosurgery of the cell wall, induction of cell fusion,^[^ ^13^ ^]^ disruption of mitochondrial functions,^[^ ^14^, ^15^, ^16^, ^17^ ^]^ and cutting of actin fibers.^[^ ^17^, ^18^ ^]^ Additionally, fs laser pulses were used to activate optogenetic proteins^[^ ^12^, ^19^ ^]^ and release small molecules into subcellular compartments. Remarkably, fs laser treatment has been demonstrated to enhance neuronal regeneration.^[^ ^20^, ^21^, ^22^ ^]^

Despite their extensive use in regulating and stimulating biological processes, fs lasers have yet to achieve their full potential due to limitations in delivery and dosage control. Conventional methods rely on static imaging followed by manual laser targeting, which is insufficient for dynamic molecular entities in live cells. These approaches lack automated target selection and flexible dosage control, making it impossible to automatically control multiple or complex molecular targets. Moreover, they separate optical control from imaging, preventing real‐time monitoring of cellular responses during fs perturbations. These limitations leave critical gaps in understanding fs laser effects on biomolecules and organelles.

Recently, real‐time precision opto‐control (RPOC) technology was developed to utilize chemically specific optical signals generated during laser scanning to direct a separate laser beam for site‐specific regulation of chemical processes. RPOC operates through a closed‐loop feedback system on a laser‐scanning microscope for fully automated, site‐specific, and chemically selective subcellular perturbation. The first RPOC was built on an SRS microscope.^[^ ^23^ ^]^ To broaden its applicability in biological research and lower system costs, a configuration using CW lasers and confocal fluorescence microscopy was developed.^[^ ^24^ ^]^ In addition, software‐assisted RPOC has significantly improved the flexibility of target selection and expanded its application potential.^[^ ^25^ ^]^ However, the action lasers currently used for chemical regulation are primarily CW lasers, which rely on linear optical processes. These linear interactions inherently limit the precision achievable in the axial (depth) dimension.

Here, we introduce fs real‐time precision opto‐control (fs‐RPOC), a technology that utilizes fs laser pulses with separately controlled average and peak power to regulate chemical processes in real time with high 3D precision. Unlike conventional methods, fs‐RPOC facilitates simultaneous optical perturbation and imaging for complex or mobile molecular targets and enables real‐time observation of cellular responses during fs laser perturbations. Compared to previous RPOC utilizing CW lasers,^[^ ^24^, ^26^ ^]^ fs‐RPOC achieves unparalleled axial precision in a 3D volume by leveraging multiphoton absorption. Additionally, a pulse‐picking method integrated with RPOC allows independent tuning of the fs laser's average and peak power, facilitating diverse, on‐demand light‐matter interaction mechanisms. Fs‐RPOC enables high‐precision single‐ and sub‐organelle microsurgery and real‐time perturbation of dynamic molecular entities, even in highly mobile systems. Applying fs‐RPOC to precisely perturb mitochondria, we uncovered distinct site‐specific cellular responses across varying laser parameters, induced by localized ROS formation, LDP generation, and delocalized H_2_O_2_ diffusion. Time‐dependent and site‐specific fluorescent signal changes reveal the intricate impact of fs laser pulses and their induced chemical stimuli on intracellular molecules.

## 2 Results

### 2.1 The Femtosecond RPOC System

**Figure 1:**
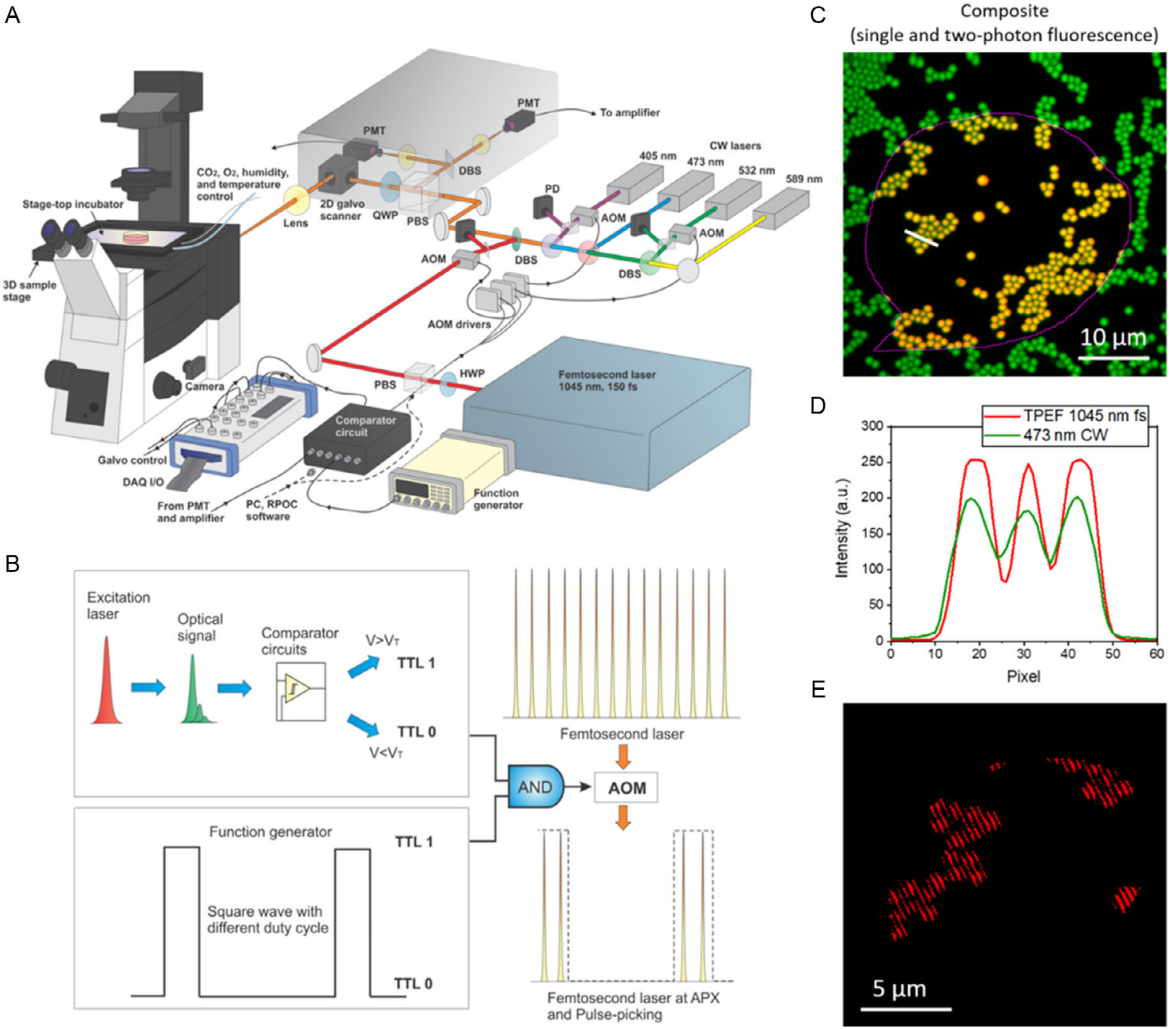
The fs‐RPOC technology. A) Schematic diagram illustrating the optical and electronic configurations of the fs‐RPOC system. Abbreviations: AOM, acousto‐optic modulator; PBS, polarizing beam splitter; PD, photodiode; DBS, dichroic beam splitter; PMT, photomultiplier tube; DAQ, data acquisition system; HWP, half‐wave plate; QWP, quarter‐wave plate. B) Conceptual illustration of achieving simultaneous RPOC and pulse‐picking using a single AOM. C) Fluorescence imaging of 1 μm fluorescent polymer particles using two modalities: two‐photon excitation fluorescence (TPEF) with 1045 nm laser pulses (red) and single‐photon excitation fluorescence with a 473 nm CW laser (green). The RPOC software was used to select ROIs for TPEF excitation with the 1045 nm laser. Yellow indicates overlapping signals from the two modalities. D) Fluorescence intensity profiles for both modalities along the line in (C). E) TPEF signals from the fluorescent microparticles acquired under 30 kHz intensity modulation of the 1045 nm fs excitation laser at a 50% duty cycle.

**Figure** **1**A shows a schematic of the fs‐RPOC system, which allows for the selection of four CW lasers for optical signal excitation and opto‐control. In this study, only the 473 and 589 nm lasers were utilized to excite fluorescence signals from fluorescent proteins or organic dyes. Fluorescence detection is carried out using two photomultiplier tubes (PMTs). These fluorescence signals, originating from biomolecules or organelles, are used to identify active pixels (APXs), defined as the pixels where action lasers are activated.^[^ ^23^ ^]^ A fs laser source serves as the major action laser, enabling the perturbation or control of biomolecular activities. The 1045 nm fs laser is directed to an acousto‐optic modulator (AOM) for simultaneous pulse‐picking and RPOC. The response time of the RPOC feedback loop, measured using a square wave input, is ≈720 ns (Figure S1, Supporting Information).

Figure 1B illustrates the concept of using the same AOM for both RPOC and pulse‐picking functionalities. The RPOC function is achieved by actively comparing optical signals from the sample to a preset threshold using a comparator circuit in real time.^[^ ^23^, ^24^ ^]^ Pulse‐picking is achieved by varying the duty cycle of a square wave sent to the AOM.^[^ ^27^ ^]^ A digital AND operation combines the comparator circuit output with the square wave, generating transistor‐transistor logic (TTL) commands for the AOM for simultaneous RPOC and pulse‐picking. In addition to relying solely on the comparator circuit, interactive RPOC software can be used to manually outline regions of interest (ROI) within the field of view (FOV). This software facilitates the simultaneous application of different optical treatment conditions or allows the selection of specific molecular targets for treatment.^[^ ^24^ ^]^

The 1045 nm fs laser operates at 80 MHz with a maximum output power of 3.5 W and a pulse width of ≈150 fs. After passing through the optical components before the microscope, the measured pulse width at 1045 nm is ≈250 fs. A square wave with a frequency of 1 MHz and a tunable duty cycle ranging from 1.33% to 97% was used for pulse‐picking. Accounting for power losses from the optical components in the system, the maximum average power and peak power that can be delivered to the sample are ≈330 mW and 16.5 kW, respectively.

To ensure precise spatial overlap of the CW excitation lasers and the 1045 nm fs laser in both lateral and axial dimensions, the platform was tested using a sample containing 1 μm fluorescent polymer microparticles. The single‐photon fluorescence signals excited by the 473 nm CW laser (green) and the two‐photon excitation fluorescence (TPEF) signals excited by the fs laser (red) showed excellent lateral overlap (yellow) (Figure 1C,D, and S2, Supporting Information). Both TPEF and single‐photon confocal fluorescence offer high axial sectioning capabilities. Figure 1C shows that the two modalities produce highly overlapping and sharp contrasts, indicating that their laser focal planes are aligned at the same axial position.

To validate the system's capability for simultaneous RPOC and pulse‐picking, a 30 kHz square wave with a 50% duty cycle was applied, while an ROI was manually selected using the RPOC software. The square wave frequency produced a distinct intensity modulation pattern within the ROI, evidenced by patterned signals and APXs from the fluorescent microparticles as shown in Figure 1E. This signal pattern results from the phase drift of the square wave along the laser scanning direction and varies with the modulation frequency, as illustrated and explained in Figure S2D–H. These results confirm the successful integration of pulse‐picking within the RPOC framework, which can enable independent control of the laser's average and peak power and facilitate the evaluation of their respective impacts on cellular responses.

### 2.2 Femtosecond RPOC Confines Laser Perturbation to the Focal Plane for Single‐ and Sub‐Organelle Microsurgery

**Figure 2:**
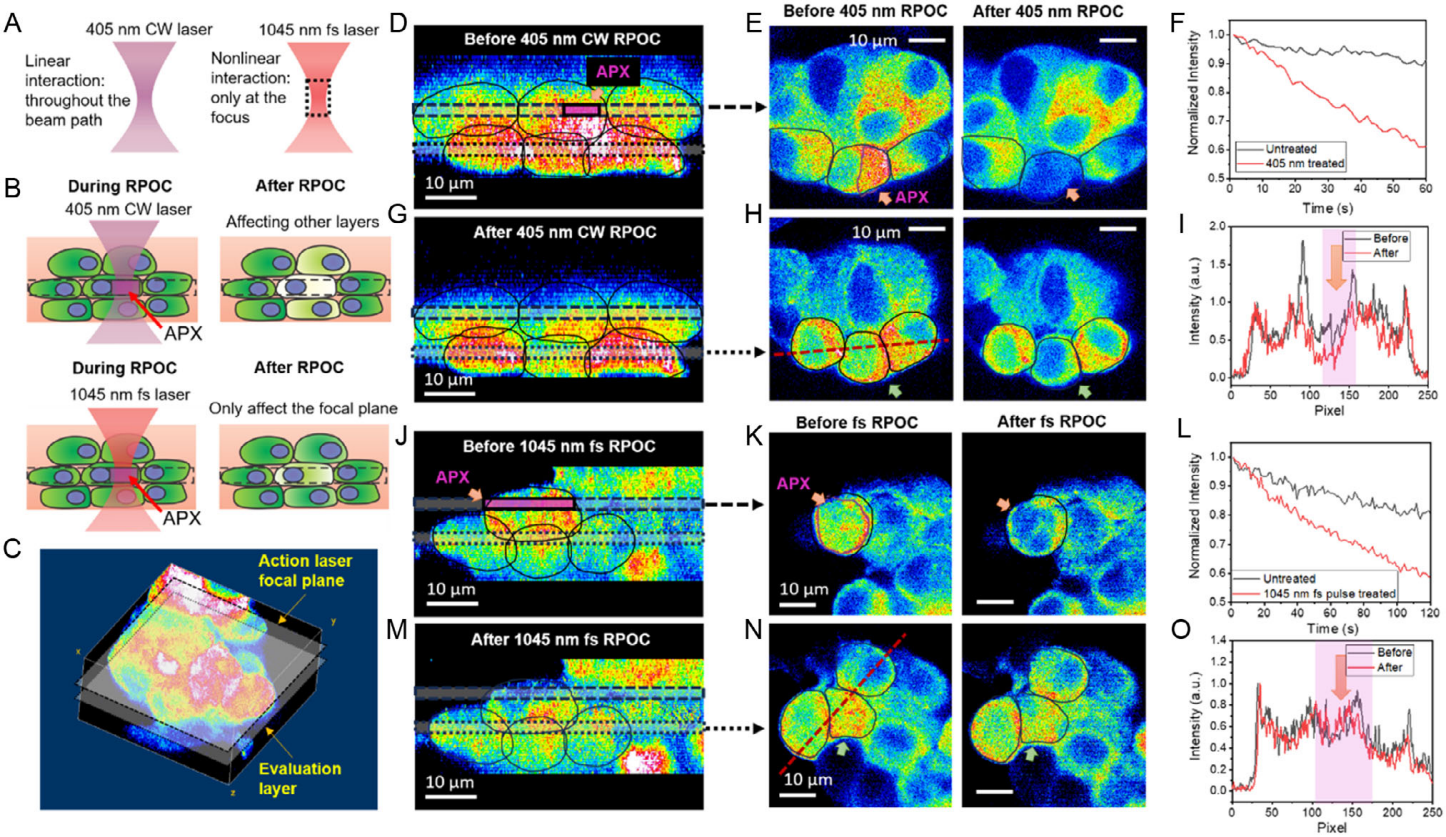
Enhanced axial precision with fs‐RPOC. A) Illustration of linear interactions occurring throughout the beam path with CW lasers versus nonlinear interactions confined to the laser focus with fs lasers. B) Schematic comparison showing extended off‐focus impact on cells using CW lasers versus minimal off‐focus impact with fs NIR lasers. C) Example of a 3D fluorescence image depicting cells, the action laser focal plane, and an evaluation plane located below the interaction focal plane. D) Side view of the 3D live HeLa cell image showing EB3‐EGFP fluorescence signals prior to treatment with a 405 nm CW laser. Cells are outlined in the images. Dashed and dotted areas indicate the action laser focal plane and the evaluation plane, respectively. E) Top view of the interaction focal plane before and after RPOC. APXs selected by the RPOC software are outlined in the image (magenta). F) Fluorescence signal decay comparison between treated and untreated cells in the same FOV. G) Side view of the 3D image showing EB3‐EGFP signals after 405 nm CW laser treatment. H) Top view of the evaluation plane before and after RPOC. I) EB3‐EGFP fluorescence signals along the dashed line from a cell (green arrow in (H)) below the treated cell (yellow arrow in (E)) before and after treatment with a 405 nm laser at 440 μW. J–O) Similar to (D)–(I), but with the selected cell treated using 13 mW fs laser pulses at 1045 nm at APXs. A negligible signal decrease was observed in the cell beneath the treated cell.

Although optical control or treatment using RPOC with CW lasers provides high spatiotemporal precision,^[^ ^24^ ^]^ the perturbation mechanism is based on linear light absorption, which can affect cells or organelles outside the focal plane. In contrast, fs laser‐based optical perturbation relies on nonlinear optical effects, which are highly localized to the laser focus where the energy density is sufficient (as illustrated in **Figure** **2**A and B). This results in enhanced 3D precision, particularly along the axial direction, while minimizing the impact on out‐of‐focus planes. To compare the effects of optical treatment using a 405 nm CW laser and a 1045 nm fs laser, we performed 3D RPOC and simultaneous fluorescence imaging of cells cultured in 3D. HeLa cells transfected with EB3‐EGFP were used to visualize cellular EB3 proteins. A focal plane was selected for laser treatment, and the impacts of RPOC on this plane and a different axial layer were evaluated (Figure 2C). The RPOC software was applied to select APXs within a cell at the interaction plane.^[^ ^24^ ^]^

When the 405 nm CW laser was applied (0.44 mW on the sample, 0.25 mJ total dose received by the cell across 68 s), the treated cell exhibited a significant loss of EB3‐EGFP signal compared to untreated controls (Figure 2D–F). The untreated cells were selected within the same imaging FOV and imaged under identical conditions as the treated cells but without exposure to the 405 nm CW laser. Notably, the 405 nm laser affected the cell beneath the treated cell (Figure 2G–I). This cell, located below the treatment laser focal plane, showed a pronounced decay in EB3‐EGFP signals after treatment (Figure 2I). In comparison, treatment with a 1045 nm fs laser (13 mW, 80 MHz, 650 W peak power, and 25.8 mJ total dose received by the cell over 120 s) also resulted in EB3‐EGFP signal decay in the treated cell within the focal plane (Figure 2J–L). However, the cell beneath the treated cell exhibited negligible changes in EB3‐EGFP signals after treatment (Figure 2M–O). These findings indicate that, compared to the 405 nm CW laser, which perturbs cells via linear absorption, the 1045 nm fs laser minimizes off‐focus perturbations, leading to reduced impact on cells outside the focal plane.

**Figure 3:**
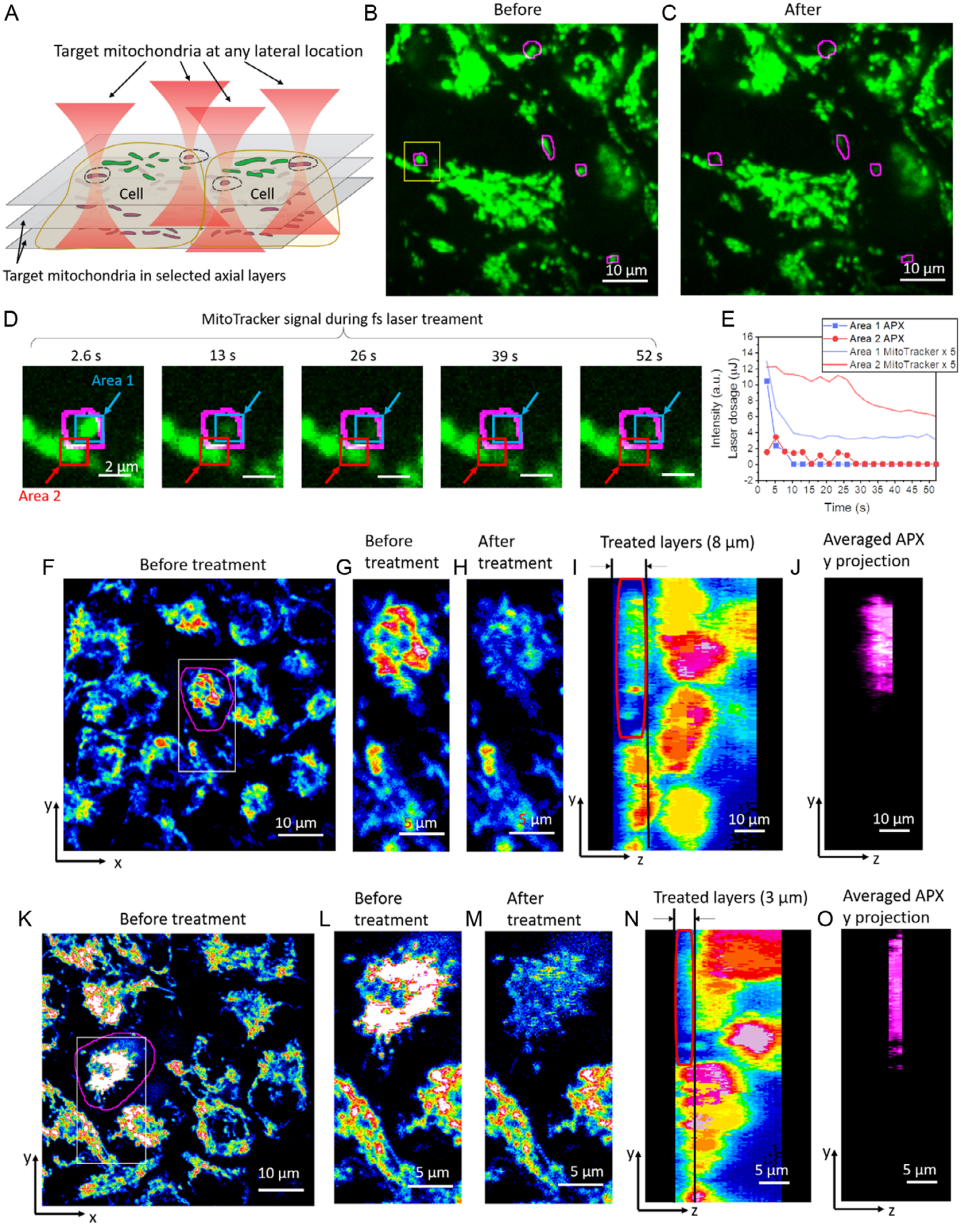
Microsurgery of mitochondria with high lateral and axial accuracy within a 3D system. A) Illustration of precise mitochondrial perturbation with single‐ or submitochondrion accuracy in both lateral and axial dimensions. B) MitoTracker signals from live HeLa cells labeled with MitoTracker. ROIs selected for fs RPOC are outlined. C) MitoTracker signals after RPOC treatment with 22 mW and 250 fs laser pulses with 1.1 kW peak power. Mitochondria within or entering the selected ROIs show significantly reduced MitoTracker signals. D) A selected area in (B) at different time points during RPOC. Area 1 indicates a fully treated mitochondrion, while Area 2 indicates a partially treated mitochondrion. E) MitoTracker signals and laser dosage calculated from APXs at Areas 1 and 2 in (D). MitoTracker signals were scaled by a factor of 5 to match the intensity range of the APX signals for displaying in the same graph. F) MitoTracker signals from HeLa cells at one interaction layer before treatment. The ROI selected by RPOC is outlined in magenta. At any axial layer for treatment, APXs within this ROI are automatically selected based on MitoTracker signals. G) Enlarged view of a selected area from (D). H) The same area as in (E) after RPOC treatment with 22 mW, 250 fs laser pulses. The treatment was performed across the bottom 8 μm of the ROI, with steps of 1 μm per frame over 8 × 5 frames (100 s). I) Side view of MitoTracker signals after treatment. The treated ROI is outlined in red. J) Side view of APXs automatically selected by fs‐RPOC within the outlined ROI. K–O) Similar to (F) and (J), but with a 3 μm layer at the bottom of the cell selected in (K) treated for 3 × 6 frames (48 s). MitoTracker signals from mitochondria within the selected volume are significantly diminished.

The high 3D spatial accuracy of fs‐RPOC enables single‐ and sub‐organelle microsurgery within a 3D volume. To demonstrate this capability, we labeled cells with MitoTracker Red and performed 3D RPOC on selected mitochondria. The results showcase both lateral and axial precision in perturbing targeted and mobile mitochondria, as illustrated in **Figure** **3**A. Using the RPOC software, we simultaneously selected five ROIs and applied a 22 mW (1.1 kW peak power) fs laser at 1045 nm, in conjunction with the comparator circuit box, to treat mitochondria within the designated ROIs. The APXs within the ROIs were automatically identified based on MitoTracker fluorescence signals and a predetermined intensity threshold.

As shown in Figure 3B,C, fs‐RPOC selectively and precisely perturbed the targeted mitochondria within the lateral plane, as evidenced by a significant reduction in MitoTracker signals following treatment. Despite the highly dynamic nature of mitochondria, RPOC enables precise targeting through automated selection of APXs. The RPOC‐selected mask and averaged APXs of this treatment are shown in Figure S3. Real‐time APXs during treatment can be found in Video S1, Supporting Information. Moreover, a mitochondrion that partially migrated into the ROIs during treatment was also affected, exhibiting noticeable MitoTracker signal loss (Figure 3D, area 2). The laser doses administered to the fully and partially treated mitochondria in Figure 3D were 15.0 and 14.4 μJ, respectively. The fully treated mitochondria received a substantially higher laser dose at the beginning (Figure 3E), resulting in a more rapid decline in MitoTracker signals. This signal decay is attributed to several factors, including multiphoton‐induced photobleaching, dye oxidation by localized ROS generation, and the leakage of MitoTracker into the cytosol due to mitochondrial membrane damage. In contrast, the partially treated mitochondria were exposed to a lower fs laser dose over an extended period, leading to a slower initial decline in MitoTracker signals (Figure 3E). However, this sequential exposure eventually triggered a more rapid signal decrease after reaching a threshold at ≈25 s, despite the termination of APXs and optical treatment at this time point. The gradual signal reduction is likely driven by dye mobility and hydrogen peroxide (H_2_O_2_) diffusion from the APX into other parts of the mitochondrion, while the accelerated decline around 25 s is likely due to mitochondrial membrane damage, causing MitoTracker to leak into the cytosol. Other ROS species, in comparison, have much shorter lifetimes and limited diffusion ranges compared to H_2_O_2_. These findings suggest that fs laser treatment targeting a small region of the mitochondrion can ultimately compromise the integrity and function of the entire organelle.

Furthermore, we applied RPOC to select APXs within a 3D cell structure attached to a culture dish (treatment over 8 μm thickness) in a 3D population. After 104 s of laser scanning, mitochondria within the targeted cell exhibited a significant reduction in MitoTracker signals (Figure 3F–J), while mitochondria in neighboring cells remained unaffected (Video S2, Supporting Information). The APXs during treatment at each axial plane and time are shown in Figure S4, which are automatically selected by RPOC based on MitoTracker signals at each layer and time point. By narrowing the treatment layer to a 3 μm thickness, as shown in Figure 3K–O, we achieved selective perturbation of mitochondria within the specified layers of the targeted cell, without disturbing other organelles in the treated area and mitochondria in other layers of the same cell.

The observed decrease in MitoTracker signals is likely attributed to a combination of two‐photon photobleaching, local ROS‐induced oxidation, and dye leakage caused by mitochondrial membrane damage. LDP formation and laser breakdown processes are unlikely to occur at the applied low peak power range. These results underscore the capability of fs RPOC to precisely target cellular organelles or other entities with high 3D spatial precision, avoiding unintended effects on neighboring locations in both lateral and axial directions.

### 2.3 Femtosecond Laser Interaction with Cells

Interactions of pulsed lasers with solid or liquid samples have been explored both theoretically and experimentally. To perform effective cellular perturbation or ablation, two schemes are typically applied. The first is using low repetition rate picosecond or nanosecond lasers, which have μJ to mJ pulse energy and perturb samples majorly through heating‐induced laser breakdown, shock wave, or intense plasma generation.^[^ ^28^, ^29^, ^30^, ^31^ ^]^ The second scheme is by applying lower energy laser pulses but with short pulse duration in the fs range at a relatively high repetition rate.^[^ ^15^, ^22^, ^32^, ^33^, ^34^ ^]^ Mechanisms involved in such fs laser interactions can range from multiphoton‐mediated electron transfer to LDP generation and ionization below the laser breakdown threshold.^[^ ^3^ ^]^ Notably, the tuning range of fs laser interaction with samples is broad, spanning from minimal perturbing power used in nonlinear optical imaging to extensive ionization and free electron generation reaching the laser breakdown level.^[^ ^3^ ^]^

From previous theoretical and experimental studies, the radiation power on the level of 10^11^–10^12^ W cm^2^ in the NIR range is found to be on the margin of generating detectable perturbations to live cells. This power range was found to generate one free electron per pulse or perturb functions of mitochondria.^[^ ^3^ ^]^

To compare our work with previous studies from other groups, we perform a calculation of the radiation in our RPOC system. Assuming the fs laser has a 33 mW average power on the sample with a 250 fs pulse duration, the pulse energy and peak power on the sample at 80 MHz are as follows

(1) $$E_{pulse}=\frac{0.033\;W}{80\;\times 10^{6}\;Hz}=0.41\;nJ$$

(2) $$P_{peak}=\frac{0.033\;W}{80\;\times 10^{6}\;Hz\times 250\;fs}\approx 1.65\;kW$$

In this work, we are using an objective lens with a 1.2 numerical aperture (NA). The focal spot size at the diffraction limit gives a diameter of 531 nm. Therefore, the laser focus has an area of about 0.22 μm^2^. The radiation power at the focus is calculated to be

(3) $$I_{peak}=\frac{1.65\;kW}{0.22{\text{ μm}}^{2}}\approx 0.75\times 10^{12}\;W/cm^{2}$$

This range is far below the laser breakdown radiation level,^[^ ^3^ ^]^ but on the margin of perturbing cell functions.^[^ ^3^ ^]^ To increase peak power while minimizing heat generation, our pulse‐picking method could separately tune the average and peak power of fs laser pulses.^[^ ^27^ ^]^ For example, when increasing the laser power to 330 mW and applying a 10% duty cycle in pulse‐picking, we could achieve 33 mW average power on the sample with a peak power of 16.5 kW. Such a peak power is close to 10^13^ W cm^2^, which was reported to induce fast mitochondrion ablation.^[^ ^3^, ^35^ ^]^ At this peak power, LDP can also potentially be generated, mediated by intrinsic biomolecules such as nicotinamide adenine dinucleotide hydrogen (NADH), flavin adenine dinucleotide (FAD), cytochrome c, or by exogenous fluorophores introduced into the cellular system such as MitoTracker. In this work, by using the pulse‐picking method, we can achieve various combinations of laser average and peak power as summarized in **Table** **1**. Owing to the use of high‐speed laser scanning in this study, we anticipate reduced photoperturbation at the same peak intensity compared to conventional methods that involve prolonged laser dwell time at a single spot. This improvement is primarily due to the significant mitigation of avalanche ionization effects.^[^ ^3^ ^]^

**Table 1:** The laser average power, duty cycle, peak power, and peak intensity applied in this study.

Average power, no pulse picking, before the microscope	Duty cycle for pulse picking	Average power on the sample (22% transmission rate)	Peak power on the sample	Peak intensity on the sample
1.5 W	10%	33 mW	16.5 kW	$7.5\times 10^{12}\;{\text{W/cm}}^{2}$
1.5 W	5%	16.5 mW	16.5 kW	$7.5\times 10^{12}\;{\text{W/cm}}^{2}$
1.5 W	1.33%	4.4 mW	16.5 kW	$7.5\times 10^{12}\;{\text{W/cm}}^{2}$
0.5 W	30%	33 mW	5.5 kW	$2.5\times 10^{12}\;{\text{W/cm}}^{2}$
0.5 W	15%	16.5 mW	5.5 kW	$2.5\times 10^{12}\;{\text{W/cm}}^{2}$
0.5 W	4%	4.4 mW	5.5 kW	$2.5\times 10^{12}\;{\text{W/cm}}^{2}$
0.1 W	75%	16.5 mW	1.1 kW	$0.5\times 10^{12}\;{\text{W/cm}}^{2}$
0.1 W	20%	4.4 mW	1.1 kW	$0.5\times 10^{12}\;{\text{W/cm}}^{2}$

### 2.4 The Dependence of Average and Peak Power of Laser Pulses

**Figure 4:**
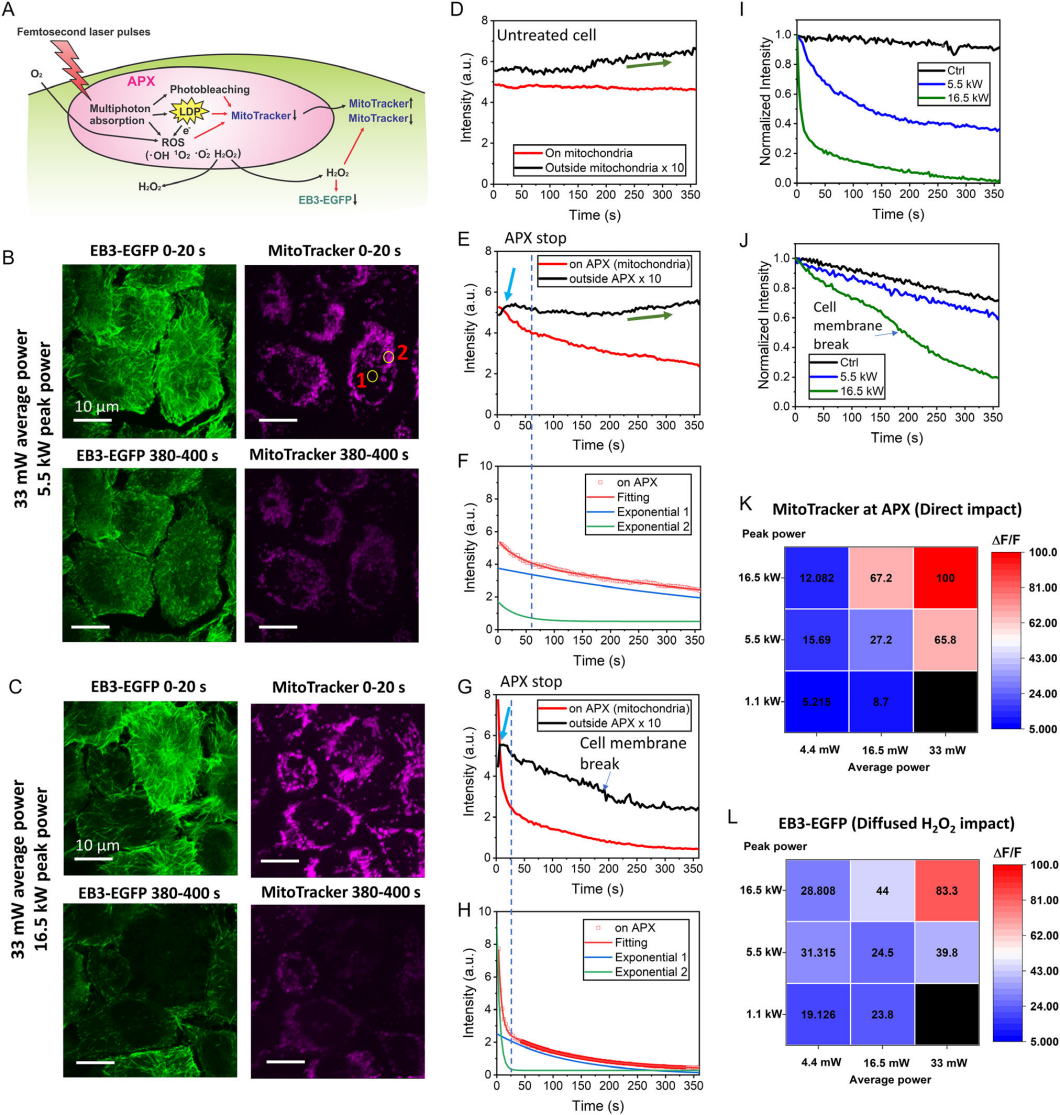
Cellular responses to fs laser treatment of mitochondria at varying average and peak power levels. A) Schematic illustration of the mechanism by which fs lasers interact with molecules within and outside APXs. B) EB3‐EGFP and MitoTracker signal at the start and end of RPOC. APXs are identified using MitoTracker signals localized to mitochondria. ROIs outside APXs (1) and within APXs (2) are selected for time‐lapse signal analysis. The fs laser pulses have 33 mW average power and 5.5 kW peak power. C) Similar to (B) but using 33 mW average power and 16.5 kW peak power fs laser pulses. D) Time‐lapse MitoTracker signals on and outside mitochondria in untreated cells. The green arrow indicates MitoTracker leaking into the cytosol. E) Time‐lapse MitoTracker signals on and outside APXs in HeLa cells from Areas 1 and 2 in (B). Signals outside APXs were scaled by a factor of 10 to allow comparison with signals on APXs using the same scale. The blue arrow indicates the initial rise of MitoTracker signals outside APXs. F) Dual‐exponential fitting of time‐lapse MitoTracker signals within APXs in (E). The dotted line marks the automatic termination time of APXs due to declining MitoTracker signals. G,H) Similar to (E) and (F), but for cells treated with 16.5 kW peak power, as shown in (C). I) Average MitoTracker signals from untreated and treated HeLa cells in (B) and (C), with peak powers of 5.5 and 16.5 kW, respectively. J) Similar to (I), but for EB3‐EGFP signals under the same conditions. K) Decay rate (ΔF/F percentage) of MitoTracker signals after RPOC using different average and peak power values. L) Decay rate (ΔF/F percentage) of EB3‐EGFP signals after RPOC using fs laser pulses with different average and peak power values.

At low average power, NIR fs lasers interact with subcellular organelles mostly through multiphoton processes. Using fs‐RPOC, we investigated the effects of varying laser power levels on cellular responses. A schematic representation of fs laser interaction with MitoTracker‐labeled mitochondria at APXs is shown in **Figure** **4**A. During laser interaction at APXs, multiphoton absorption—primarily facilitated by MitoTracker and/or intrinsic biomolecules—can lead to photobleaching of MitoTracker by directly altering the dye's structure, independent of molecular oxygen. Additionally, this absorption may promote a triplet state of the fluorophore that is quenched by molecular oxygen, generating ROS. The resulting local ROS oxidation of MitoTracker further diminishes its fluorescence signal. Under very high peak power conditions, multiphoton absorption can also lead to the formation of LDPs, which both directly degrade MitoTracker molecules or indirectly contribute to their damage via LDP‐induced ROS.

Damage to mitochondrial membranes caused by these mechanisms can lead to the leakage of MitoTracker from mitochondria, increasing MitoTracker signals in the vicinity of the APXs. Additionally, H_2_O_2_, a type of ROS generated at the APXs, can diffuse from the mitochondria into the cytosol.^[^ ^36^ ^]^ In contrast, other ROS species such as singlet oxygen, hydroxyl radicals, and superoxide ions are less likely to diffuse significantly beyond the APXs.^[^ ^37^, ^38^ ^]^ The diffused H_2_O_2_ can oxidize both EB3‐EGFP and the leaked MitoTracker outside the APXs.^[^ ^39^ ^]^ The competing effect of MitoTracker leakage from APXs and its oxidation by H_2_O_2_ could lead to varying changes in MitoTracker signal outside APXs over time.

The efficiency of multiphoton processes depends on laser peak power levels, leading to significant variations in fs laser‐induced interactions at APXs and the resulting cellular responses. To explore these differences, we employed fs‐RPOC with pulse‐picking to independently control laser average and peak power, targeting mitochondria in EB3‐EGFP‐transfected HeLa cells. APXs were automatically selected based on MitoTracker signals using comparator circuits of the RPOC system. The use of comparator circuits enables real‐time dynamic comparison of MitoTracker signals against a predefined threshold. As the MitoTracker signals decay during treatment, the number of APXs and treatment laser dose typically decrease over time. The treatment automatically terminates once the MitoTracker signals fall below the threshold. Changes in EB3‐EGFP signals primarily reflect the impact of H_2_O_2_ released from APXs, while MitoTracker signals primarily indicate both direct laser interactions and indirect effects of ROS (Figure 4B–J). We monitor both effects using separate fluorescence channels.

Using pulse‐picking, we first achieved an average power of 33 mW for 1045 nm laser pulses, with a peak power of 5.5 kW at APXs. Over a 380‐s RPOC period, EB3 comets exhibited significant shortening (Figure 4B and Video S3, Supporting Information), indicating strong phototoxicity induced by fs laser pulses at APXs.^[^ ^26^ ^]^ When the peak power was increased to 16.5 kW while maintaining the same 33 mW average power, the fs laser treatment caused more severe photodamage, characterized by membrane rupture and the complete loss of intracellular EB3‐EGFP and MitoTracker signals (Figure 4C and Video S4, Supporting Information).

To quantify these effects, we first monitored MitoTracker signals on and outside mitochondria in untreated cells as a control. On mitochondria, MitoTracker signals remained stable over 380 s of imaging (Figure 4D). Outside mitochondria, signals showed a slow increase, likely due to MitoTracker leakage induced by the 473 nm imaging laser (Figure 4D).

We selected areas (e.g., Figure 4B, areas 1 and 2) at APXs and outside APXs to understand the direct fs laser impact and the indirect MitoTracker leaking and oxidation by H_2_O_2_. In cells treated with 33 mW average power and 5.5 kW peak power, MitoTracker signals at APXs show a decay (Figure 4E), highlighting the direct effects of fs laser pulses. This decay followed a two‐phase pattern (Figure 4F): an initial rapid decline due to APX activity, ceasing around 20 s as MitoTracker signals fell below the APX threshold, followed by a slower decay caused by ROS oxidation and MitoTracker leakage from mitochondria. Outside APXs, MitoTracker signals initially rose rapidly, indicating MitoTracker leakage into the cytosol, then declined due to H_2_O_2_ diffusion and oxidation of leaked MitoTracker (Figure 4E). Once the fs laser at APXs was automatically deactivated, the production of ROS stopped, leading to partial recovery of MitoTracker signals outside APXs due to the continued dye leakage, similar to the untreated cells (Figure 4D).

Increasing the fs laser peak power to 16.5 kW (while keeping the same 33 mW average power) significantly amplified these effects. In the MitoTracker channel, APX signals decayed rapidly during laser activation, followed by a slower decay postdeactivation (Figure 4G), both well fitted by two‐phase exponential functions (Figure 4H). This decay occurred more quickly than in the 5.5 kW peak power case, with MitoTracker signals dropping below 15%, indicating extensive molecular damage at APXs. Outside APXs, signals initially spiked before declining more rapidly than in the 5.5 kW case due to higher H_2_O_2_ production and leakage. Unlike the 5.5 kW case, which exhibited a partial increase in the MitoTracker signal, the 16.5 kW peak power case showed continued signal decay outside APXs even after APX deactivation, suggesting elevated cytosolic H_2_O_2_ levels. Strong damage to MitoTracker at APXs by 16.5 kW peak power led to significantly lower MitoTracker levels in mitochondria, preventing further MitoTracker signal rise due to leakage. Furthermore, around 170 s, the rupture of the cell membrane further accelerated MitoTracker leakage outside of the cell (Figure 4G). The averaged MitoTracker signals change over time for both treatment conditions, along with the control, are compared in Figure 4I.

In the EB3‐EGFP channel, treatment with 5.5 kW peak power resulted in less signal decay compared to 16.5 kW (Figure 4J), confirming greater H_2_O_2_ generation at higher peak power. Cell membrane rupture was also detectable as the accelerated EB3 signal loss at around 170 s, consistent with the MitoTracker response in Figure 4G.

The distinct fluorescent signal changes observed at different laser peak power levels demonstrate that fs laser‐induced perturbations to mitochondria and cellular functions are highly peak power‐dependent. Varying peak power levels result in differences in the rates of molecular damage at APXs, leakage from APXs, and oxidation outside APXs. These effects are reflected in the fluorescence signal dynamics over time.

To further assess the impact of fs laser average and peak power on mitochondrial‐targeted cellular responses, we compared the fluorescence signal decay rates (ΔF/F) in the MitoTracker and EB3‐EGFP channels across treated cells over 380 s using different combinations of average and peak power (Figure 4K,L). At 5.5 kW peak power, significant effects emerged when the average power reached 33 mW, whereas at 16.5 kW peak power, notable impacts were observed at average powers above 16.5 mW. These results indicate that at 16.5 mW average power and above, the cellular response is peak power dependent. Consequently, the decline in cellular fluorescence signals under high peak power fs laser pulses is unlikely to result from thermal effects due to linear absorption.

This conclusion is further supported by experiments illuminating the entire FOVs with similar average power but much lower peak power, as shown in Figure S5. Compared to the control group (no fs laser applied), illuminating the entire FOV over 320 s with laser average powers of 11 or 22 mW and peak powers ranging from 343 to 688 W resulted in minimal EB3‐EGFP signal decay. Higher peak power only slightly enhances the shortening of EB3 comets (Video S5, Supporting Information). When centrosomes were present within the FOV and illuminated by fs lasers, the corresponding cells showed slightly enhanced shrinkage. These findings further confirm that at high peak power, fs laser‐induced perturbations to cellular functions are primarily due to multiphoton processes rather than linear absorption of NIR fs laser pulses.

The results in Figure 4 indicate that cellular responses depend on both peak and average power. While high peak power is essential to enable efficient multiphoton absorption, the generated ROS or LDP must surpass a critical threshold to elicit significant cellular effects. At lower concentrations, ROS or LDP produced through multiphoton processes at APXs do not induce noticeable cellular responses within the observed timeframe. Future studies will explore long‐term cellular responses under varying laser power levels and dosages.

### 2.5 The Involvement of ROS

**Figure 5:**
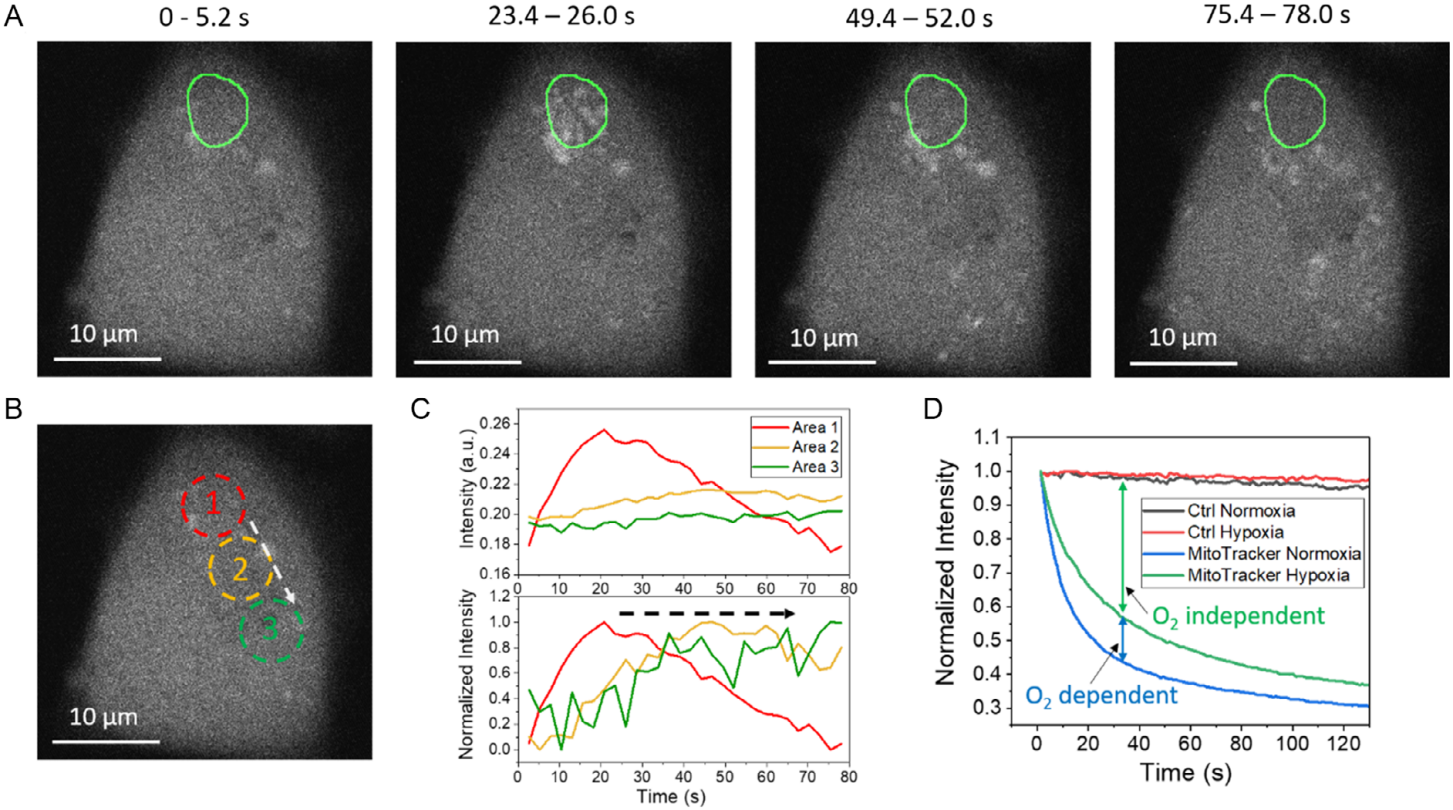
Confirming the involvement of molecular oxygen and ROS during fs‐RPOC. A) Time‐lapse images of H2DCFDA fluorescence in MIA PaCa‐2 cells following excitation with 110 mW, 1045 nm fs laser pulses at the designated area (outlined in green) by RPOC. B) Regions selected for quantitative analysis of H_2_O_2_ propagation. C) Time‐lapse plots of absolute fluorescence intensity (top) and normalized intensity (bottom) of H2DCFDA in the three color‐coded regions shown in (B). The arrow indicates H_2_O_2_ diffusion direction from area 1 to area 3. D) Changes in MitoTracker Red fluorescence over time during fs‐RPOC under normoxic and hypoxic conditions. Control groups (ctrl) represent cells imaged without exposure to fs laser pulses. Molecular oxygen‐dependent and independent components of the responses are indicated in the graph.

To demonstrate the involvement of ROS in fs‐RPOC treatment of cells, we used a fluorescent turn‐on ROS sensor, dichlorodihydrofluorescein diacetate (H2DCFDA).^[^ ^26^ ^]^ MIA PaCa‐2 cells were co‐stained with H2DCFDA and MitoTracker Red. A specific region enriched with mitochondria was selected and treated using fs‐RPOC with laser pulses at 110 mW average power and 5.5 kW peak power. As shown in **Figure** **5**A, ROS were detected in the treated area, and their subsequent propagation was visualized within 78 s (Video S6 and S7, Supporting Information). Upon extensive ROS accumulation, the H2DCFDA signal eventually decayed, due to dye degradation (Figure 5B,C). Among the various ROS species, only hydrogen peroxide (H_2_O_2_) is expected to diffuse over several micrometers and throughout the cell, contributing to the observed spatial spread of the signal in Figure 5A–C.

To further confirm the role of molecular oxygen in this process, we compared MitoTracker signal decay following fs‐RPOC treatment under normoxic and hypoxic conditions (Figure 5D). For the hypoxia experiment, cells were incubated in 0.1% oxygen with a stage‐top incubator for 4 h before laser treatment. Using identical laser intensities and exposure times, we observed a more significant reduction in MitoTracker Red fluorescence under normoxia. This result supports the involvement of molecular oxygen and ROS in MitoTracker oxidation, consistent with previous reports of ROS generation under single‐photon excitation mediated by MitoTracker Red absorption.^[^ ^26^ ^]^ Notably, even under hypoxia, MitoTracker fluorescence still decreased upon fs‐laser treatment, indicating a component of direct photobleaching through multiphoton absorption that occurs independently of molecular oxygen (Figure 5D).

### 2.6 The Involvement of LDP

In Figure 4A, LDP may be induced by multiphoton absorption of fs lasers at high peak power. These LDP are highly reactive and likely interact with surrounding molecules instantaneously. The LDP is observable through white light generation only when their concentration reaches a sufficient threshold to the avalanche ionization level.^[^ ^3^ ^]^ When using fs‐RPOC to target and perturb mitochondria, we did not observe white light generation due to the MitoTracker signal decay which rapidly turned off APXs and fs laser pulses.

**Figure 6:**
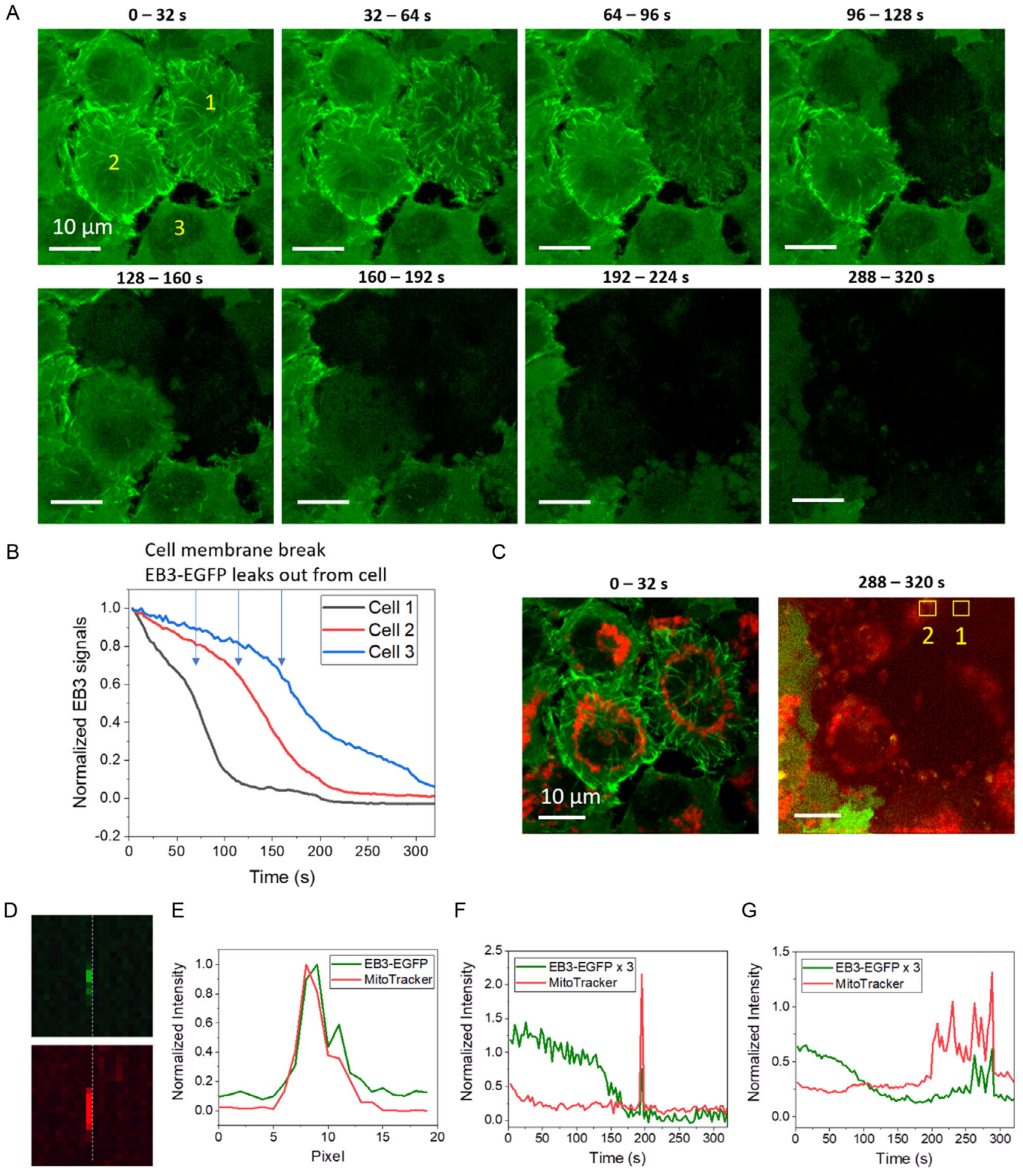
Confirming LDP involvement through white light generation. A) EB3‐EGFP signals from HeLa cells during RPOC treatment with 16.5 mW average power and 16.5 kW peak power. The fs laser is activated across the entire FOV. Three cells are labeled as 1,2,3 for comparsion. B) EB3‐EGFP signal decay over treatment time for three selected cells in (A). Arrows indicate time points where plasma membrane rupture occurs. C) Correlated EB3‐EGFP and MitoTracker images from the same FOV as (A), captured at the beginning and end of RPOC. Example areas of white light generation are outlined in yellow squares. D) White light emission detected in both EGFP and MitoTracker channels from Area 1 in (C) at 61 s. E) Intensity profiles of the white light signals in (D). F) Time‐lapse intensity within selected Area 1 from (C) in both fluorescence channels. G) Time‐lapse intensity within selected Area 2 from (C).

To confirm the involvement of LDP, we turned the action laser on throughout the entire frame using fs laser pulses with 16.5 mW average power and 16.5 kW peak power and treated the cells for 320 s. This resulted in significantly more pronounced cell damage (**Figure** **6**A) compared to previous cases. Membrane rupture of cell 1 occurred at ≈64 s. Initially, EB3 comets remained intact, followed by a rapid decline in fluorescence signals and eventual plasma membrane rupture (Figure 6A). This cell death process differs from the mitochondria‐targeted case shown in Figure 4B. It shows no shortening of EB3 comets but rapid bleaching of EB3‐EGFP, followed by membrane rupture and leakage of cellular contents (Video S8, Supporting Information).

Additionally, due to the slight laser energy inhomogeneity within the FOV, cells exhibited photo‐damage at slightly different rates. All three selected cells in the image showed membrane rupture at different time points, characterized by accelerated EB3‐EGFP signal loss (Figure 6B) similar to observations in Figure 4J. During these damage processes, white light generation was observed (Figure 6C–G), with significantly stronger signals than fluorescence, detectable in both the EGFP and MitoTracker channels. These emissions show sharp spikes, originating from LDP‐induced white light generation, and have strong spatial and temporal correlation across both fluorescence channels (Figure 6D–G).

These findings indicate that 1045 nm fs laser pulses at 16.5 kW peak power can induce LDP. The rate of LDP generation and its impact on cells are dosage‐dependent. At low LDP dosages, cellular functions remain largely unaffected, as shown in Figure 4K,L.

## 3 Discussion

Fs‐RPOC offers optimal spatiotemporal precision, particularly in the axial direction, to perturb biomolecular functions accurately. The integration of a pulse‐picking mechanism into RPOC enables independent control of pulse average and peak power at APXs, allowing precise modulation of cellular targets with separately adjustable average and peak power levels. We demonstrated the precise targeting of selected mitochondria in a 3D system and evaluated the effects of varying average and peak power on cellular responses during mitochondrial perturbation. Different power levels elicited diverse cellular responses due to variations in the production of ROS, LDP, and molecular diffusion within cells.

It is worth noting that although fs laser pulses offer higher axial spatial precision for optical perturbation compared to visible lasers, they are less efficient at exciting EGFP signals than 473 or 488 nm CW lasers. A direct comparison between single‐photon fluorescence and nondegenerate TPEF of EB3‐EGFP signals is shown in Figures S5 and S6. Under TPEF, high‐energy fs pulses (30 mW total) do not produce signals comparable to those generated by a 473 nm CW laser at 450 μW. TPEF imaging does not clearly resolve EB3‐EGFP comets and induces much stronger photobleaching compared to 473 nm CW laser excitation. EB3‐EGFP signals are effective readouts of cellular responses to perturbation, and single‐photon excitation is required for their reliable visualization.

In this study, we utilized 1045 nm fs laser pulses for cellular organelle perturbation. Our system also includes a frequency‐tunable fs laser, which can be employed as an action laser for RPOC. This capability would enable wavelength‐dependent evaluations of site‐specific perturbations to cellular functions and provide insights into laser interaction mechanisms by comparing wavelength‐dependent responses with the absorption spectra of specific molecules. Future studies will explore these wavelength‐dependent effects.

This study presents an advanced system for controlling and perturbing cellular targets with fs lasers, offering flexible combinations of average and peak power. We demonstrate single‐ and sub‐organelle microsurgery in live cells with high spatial precision and simultaneous monitoring of cell responses. Additionally, our findings provide new insights into how site‐specific fs laser perturbations affect cellular responses through various multiphoton‐induced processes at different power levels. By advancing our ability to perturb biomolecules with fs laser pulses at high spatial precision and simultaneous imaging, fs‐RPOC opens new avenues for understanding local or global cell responses to site‐specific molecular activities.

## 4 Experimental Section

4.1

4.1.1

##### The fs‐RPOC Technology

The fs‐RPOC system is based on a custom‐designed confocal fluorescence microscope. It incorporates four CW lasers (405, 473, 532, and 589 nm) for fluorescence excitation and optical control. The 405 and 532 nm lasers are used as optional CW action lasers. AOMs (P80L‐0.5 with 532B‐2 driver, Isomet) are installed in the 405 and 532 nm laser paths, functioning as fast optical shutters. These lasers are coupled to the sample via the first‐order diffraction. The beams are combined collinearly using dichroic beam splitters (DMLP567, DMLP505, and DMLP425, Thorlabs), with variable neutral density filters in each path for laser power attenuation.

The 1045 nm fs laser (InSight X3+, Spectral Physics) passes through another AOM for simultaneous RPOC and pulse‐picking. All lasers are directed to the microscope via a 2D galvo scanner (Saturn‐5 system, ScannerMAX), expanded to 9 mm by a lens pair, and focused through a water‐immersed objective lens (UPLSAPO‐S, 60X, NA 1.20, Olympus) mounted on an inverted microscope (Olympus IX73). TPEF images in Figure 1 are acquired using the fs action laser by keeping the laser constantly on using the comparator circuit box.^[^ ^25^ ^]^ In Figure S5, to achieve varying peak power without pulse‐picking, a 150 mm SF‐75 glass rod was introduced into the 1045 nm fs laser beam path to chirp the pulses as needed.

Fluorescence signals are separated using a polarizing beam splitter and a quarter‐wave plate, then detected by two PMTs (H7422‐40, Hamamatsu) through confocal pinholes (P300HK, Thorlabs). Filters enable the detection of EGFP and organelle dyes in separate channels. Preamplifiers (PMT4V3, Advanced Research Instruments Corporation) convert and amplify signals for data acquisition (PCIe‐6363, National Instruments). A comparator circuit, previously reported,^[^ ^25^ ^]^ automates APX selection. The feedback loop for fs‐RPOC has a response time of ≈720 ns (Figure S1, Supporting Information).

Pulse‐picking is achieved with RPOC by real‐time computation of RPOC TTL with a square wave from a function generator (DG1022Z, Rigol). The frequency and duty cycle of the square wave are tunable. The comparator circuit applies a digital AND operation to combine (multiply) the RPOC output with the square wave.

The RPOC software, developed in LabVIEW, integrates laser control and imaging.^[^ ^25^ ^]^ It allows manual ROI delineation, automatic target selection based on optical signals, and precise targeting of dynamic molecular entities within the ROI. The software supports 3D RPOC and imaging across the ROI or the full FOV. APXs are monitored using photodiodes (PDA10A2, Thorlabs) which detect small fractions of the action lasers deflected by glass slides.

A stage‐top incubator (WSKMX with STX‐CO2O2, Tokai Hit) maintains precise humidity, temperature, CO_2_, and O_2_ levels for cultured cells, enabling continuous monitoring of the same FOV for over 48 h without changes of culture medium.

##### RPOC, Imaging, and Data Processing

For cells in Figure 2D–H, a 3D image acquisition with a thickness of 30 μm was performed prior to the RPOC treatment. An ROI within a cell in the interaction layer was selected using the RPOC software. The treatment was applied by scanning the 405 nm laser (440 μW on the sample) for 68 s consecutively at this layer within APXs, with simultaneous image acquisition during the process. The outlined areas of APX received 0.25 mJ total laser dosage. After the treatment, another 3D acquisition was conducted to compare pre‐ and post‐treatment signals. Time‐dependent intensity profiles were generated by integrating EB3 fluorescence signals from each frame during treatment and plotted using Origin 2021. The cells in Figure 2J–N were imaged under similar conditions as those in Figure 2D–H. The fs laser treatment was conducted using a 1045 nm fs laser with a pulse width of 250 fs for 120 s. The APX area received a total fs laser dose of 25.8 mJ. Imaging was performed at 1.3 frames per second with a pixel dwell time of 2.5 μs.

Intensity profiles from cells in Figure 2 were compared along the same line at the same layers before and after treatment. All 3D images were processed in ImageJ and displayed using a Jet color scheme.

In Figure 3B, C, separate ROIs were selected using the RPOC software. Comparator circuits were used in combination with the RPOC software to control dynamic mitochondria within the selected area. MitoTracker signals were used to identify real‐time APXs through the comparator circuit. Treatments were applied by scanning the laser for 52 s. In Figure 3F–O, RPOC treatment was continuously executed within the selected layer of the 3D cell structure for different time lengths indicated in the figure caption. 3D images were acquired following the same procedure as in Figure 2. Images were processed in ImageJ to display *x–y* and *y–z* projections, and 3D APXs were visualized in the *y–z* plane.

In Figures 4, 5, 6, intensity plots for APX regions and areas outside of APXs were generated using ImageJ and subsequently graphed in Origin 2021. Curve fitting with single or dual exponential functions was performed in Origin 2021. Heatmap plots showing the percentage of fluorescence signal decay in each channel after treatment were generated and visualized using Origin 2021.

##### Cell Preparation and Labeling

HeLa Kyoto EB3‐EGFP cells were purchased from Biohippo and cultured in Dulbecco's Modified Eagle Medium (DMEM, ATCC) supplemented with 10% fetal bovine serum (FBS, ATCC) and 1% penicillin/streptomycin (Thermo Fisher Scientific). The cells were seeded into glass‐bottom dishes (MatTek Life Sciences) containing 2 mL of culture medium and incubated in a CO_2_ incubator at 37 °C with 5% CO_2_. Cells were grown overnight until they reached ≈50%–70% confluence for live‐cell imaging and RPOC. MiaPaCa‐2 cells were obtained from ATCC and prepared similarly.

For labeling mitochondria, the cells were incubated with MitoTracker Red CMXRos at a final concentration of 200 nM. After a 30‐min incubation at 37 °C and 5% CO_2_, the cells were gently rinsed twice with a warm culture medium before imaging and RPOC.

For ROS measurement, dichlorodihydrofluorescein diacetate (H2DCFDA, Thermo Fisher Scientific) was added to the culture medium at a final concentration of 10 μM, and MitoTracker Red CMXRos was applied at 200 nM. The experiment was conducted under both normoxia and hypoxia (0.1% O_2_ and 5% CO_2_) to compare the involvement of molecular oxygen and the generation of ROS. The stock solution of H2DCFDA has a concentration of 20 mM and was prepared inside a nitrogen‐purged glove box to avoid degradation. For the ROS study in Figure 5, both H2DCFDA and MitoTracker Red CMXRos were simultaneously added to the cells, which were then incubated for 30 min at 37 °C containing 5% CO_2_. Before imaging, dye‐labeled cells were rinsed with the prewarmed culture medium. For the normoxia condition, cells were imaged and treated in a stage‐top incubator maintained at 19% O_2_ and 5% CO_2_. For the hypoxia condition, rinsed cells were incubated in 0.1% O_2_ and 5% CO_2_ for 4 h before RPOC treatment and imaging.

## Conflict of Interest

Dr. Chi Zhang is the founder of Photokinesis LLC, a startup company that aims to commercialize advanced opto‐control technologies. Other authors declare no conflict of interest.

## Author Contributions

**Seohee Ma:** data curation (lead); formal analysis (lead); visualization (lead); writing—original draft (supporting). **Bin Dong:** data curation (supporting); formal analysis (supporting); validation (equal). **Matthew G. Clark:** data curation (supporting). **R. Michael Everly:** software (lead). **Shivam Mahapatra:** data curation (supporting); formal analysis (supporting). **Chi Zhang:** conceptualization (lead); data curation (supporting); funding acquisition (lead); supervision (lead).

## Supporting information

Supplementary Material

## Data Availability

All data supporting the findings of this study are included in the main text and Supporting Information (SI) and can be requested from the corresponding author upon reasonable request.
