# Supplementary material for: Real‐Time and Site‐Specific Perturbation of Dynamic Subcellular Compartments Using Femtosecond Pulses
Source: Small Sci. 2025 May 22;5(7):2500166. doi: 10.1002/smsc.202500166 (PMC12257886; doi:10.1002/smsc.202500166)
Supplement: Supplementary file 1 — Supplementary Material [file SMSC-5-2500166-s001.zip › smsc202500166-sup-0001-SuppData-S1.pdf]

Supporting information

Real-Time and Site-Specific Perturbation of Dynamic Subcellular Compartments Using Femtosecond Pulses

Seohee Ma[1,2], Bin Dong[1,2], Matthew G. Clark[1], R. Michael Everly[1], Shivam Mahapatra[1,2], Chi Zhang[1,2,3]

[1]James Tarpo Jr. and Margaret Tarpo Department of Chemistry, Purdue University, 560 Oval Dr., West Lafayette, IN 47907, USA

[2]Purdue Institute for Cancer Research, Purdue University, 201 S. University St., West Lafayette, IN 47907, USA.

[3]Purdue Institute of Inflammation, Immunology, and Infectious Disease, Purdue University, 207 S. Martin Jischke Dr., West Lafayette, IN 47907, USA.

*zhan2017@purdue.edu

Supporting Figures

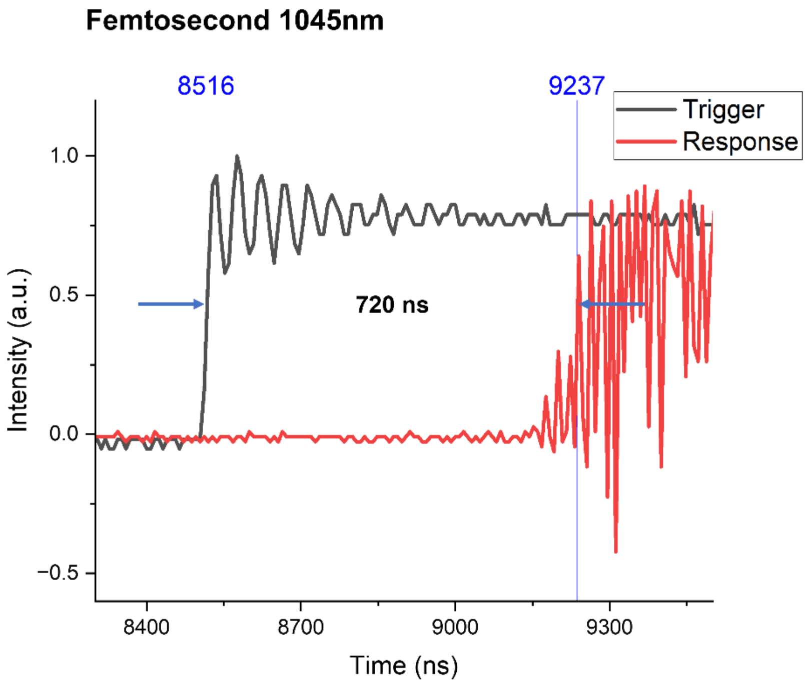

Figure S1. The response time of the fs-RPOC system measured using a square wave input command (black) and photodiode detection of the fs lasers after deflection by an AOM (red).

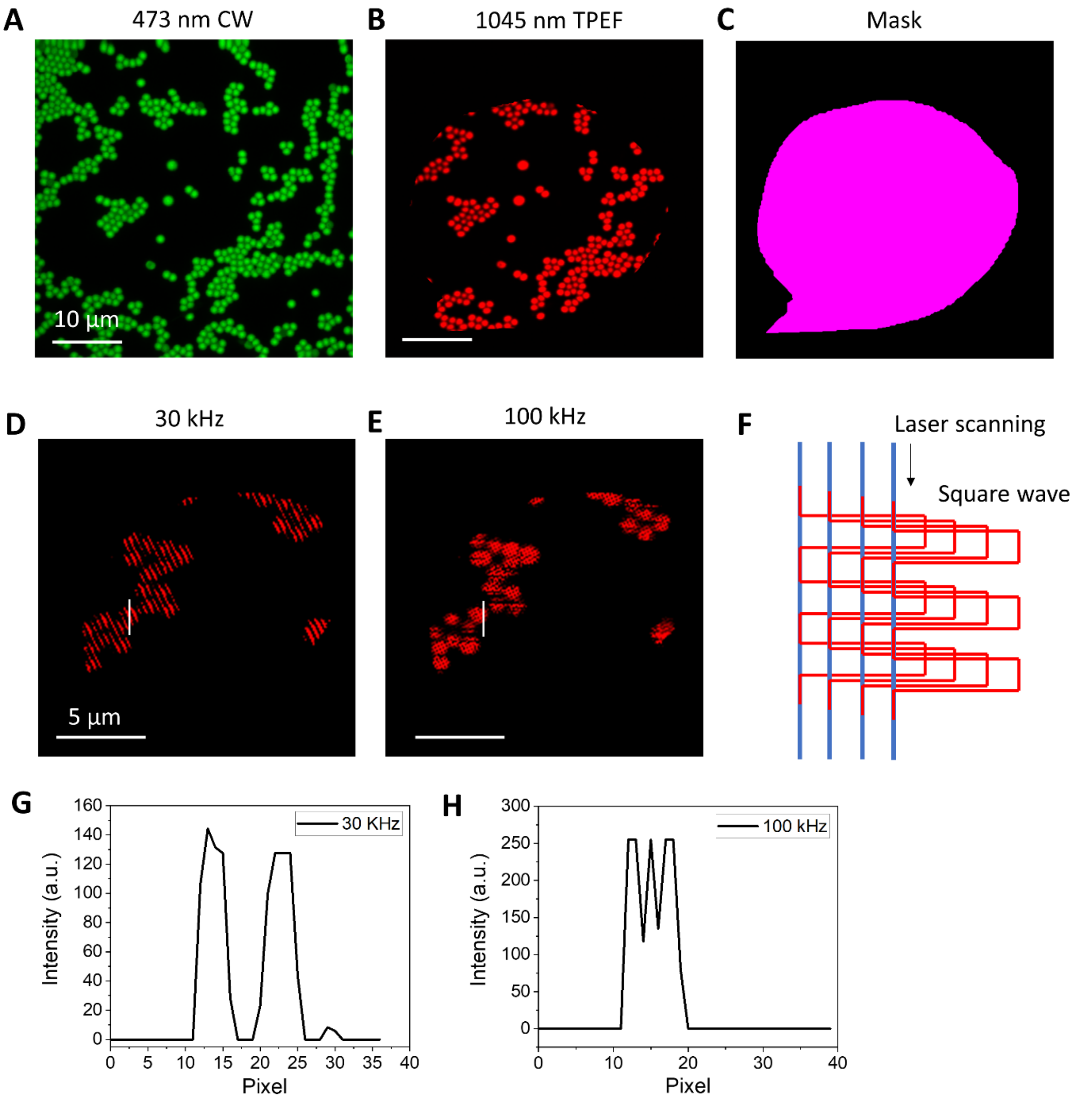

Figure S2. Characterization of the fs-RPOC system. A) Fluorescent microparticles (1 µm in diameter) excited by a 473 nm CW laser. B) The same field of view as in (A), with fluorescent microparticles excited by a 1045 nm fs laser via two-photon absorption. The ROI was selected using RPOC software. C) RPOC software-generated mask used for TPEF imaging and RPOC testing. The fluorescence signals in (A) and (B) are detected from the same signal channel. D) TPEF signals from fluorescent microparticles generated using 30 kHz square wave modulation (50% duty cycle) of the excitation fs laser. E) Same as (D), but with 100 kHz modulation. F) Illustration of signal stripe formation resulting from low-frequency modulation of the excitation laser; the stripe spacing depends on the modulation frequency. G) TPEF intensity profile along the solid line in (D). H) Simiar to (G), but along the solid line in (E).

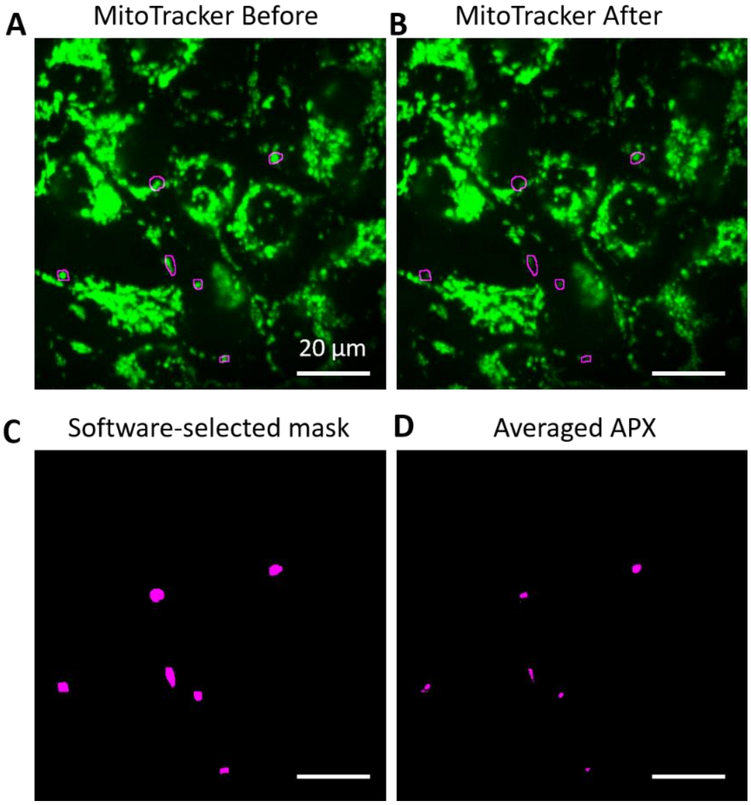

Figure S3. fs-RPOC enabled single- and sub-organelle microsurgery. A) and B), MitoTracker signals before and after RPOC. The RPOC-software-selected ROIs are outlined in magenta. C) RPOC software-selected ROIs. D) Averaged APX, which are automatically selected by comparator circuits within the chosen ROIs.

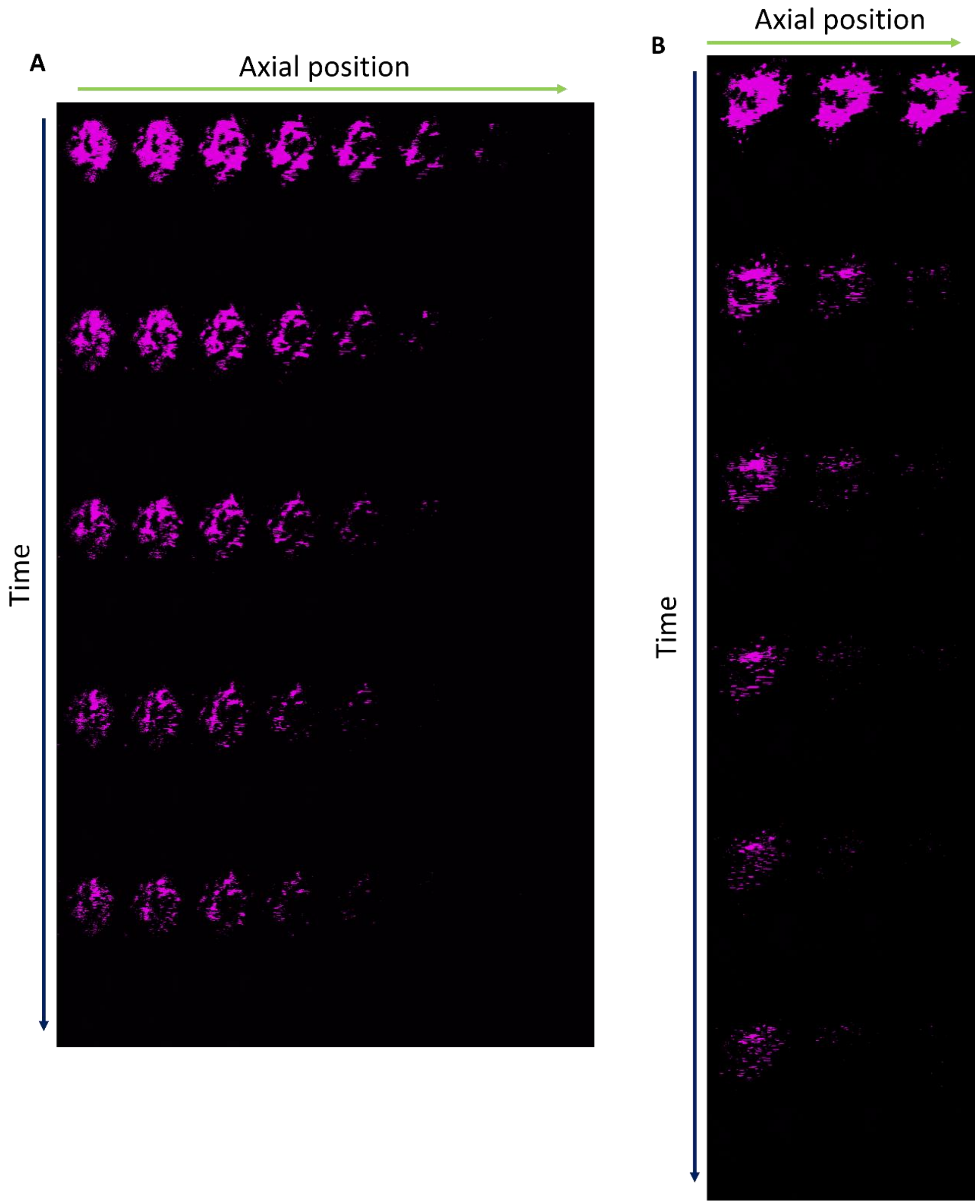

Figure S4. APX across different depths and treatment durations corresponding to Figure 3. A) APXs for the treatment of the 8 μm layer shown in Figures 3F–J. A total of 8 layers in axial direction are scanned at each time point for 5 time points. The APX images are cropped from the original image similar to Figure 3G. B) APXs for the treatment of the 3 μm layer shown in Figures 3K–O. A total of 3 layers in axial direction are scanned at each time point for 6 time points. The APX images are cropped from the original image similar to Figure 3L. Fs-RPOC treatment is performed at 2.6 s per frame.

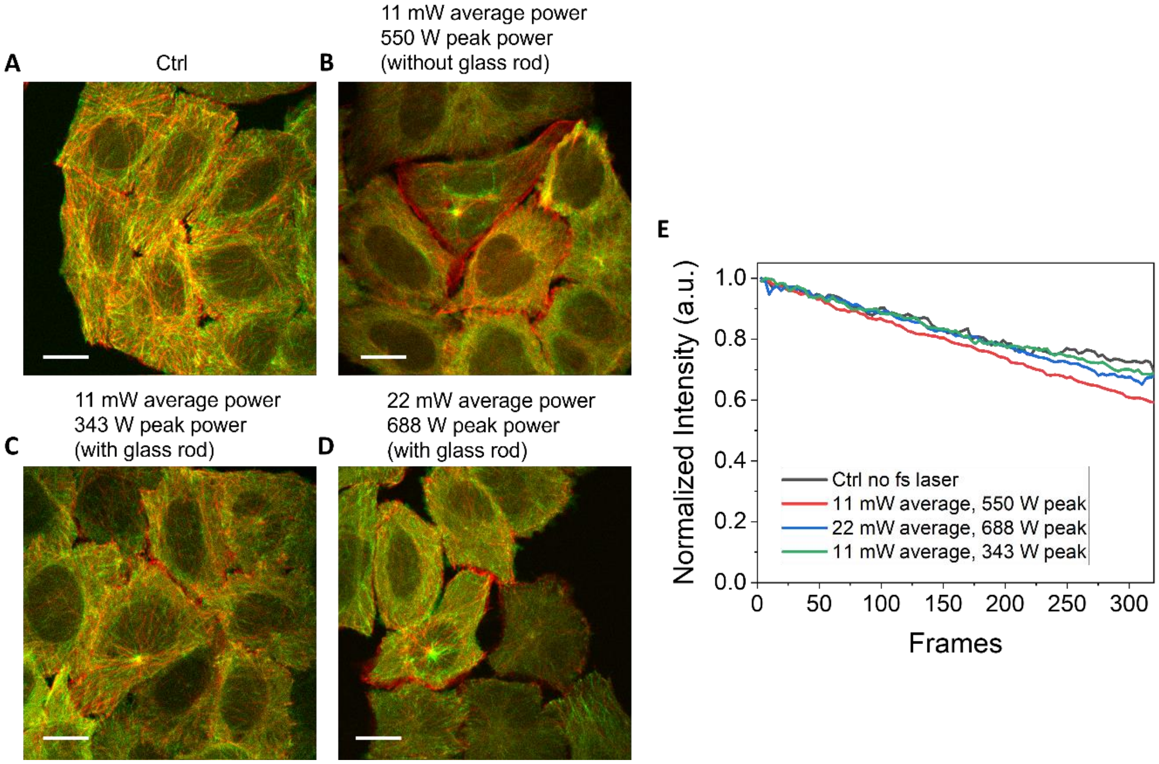

Figure S5. Interaction of lower peak power 1045 nm fs laser with cells. A) EB3-EGFP cells imaged with a 450 μW 473 nm laser for 320 s. The red and green contrasts represent the averaged images from the first and last five frames, respectively. B–D) Similar to (A), but with cells exposed to different average and peak powers of a 1045 nm fs laser. E) Averaged EB3-EGFP signals from cells under different conditions shown in (A–D).

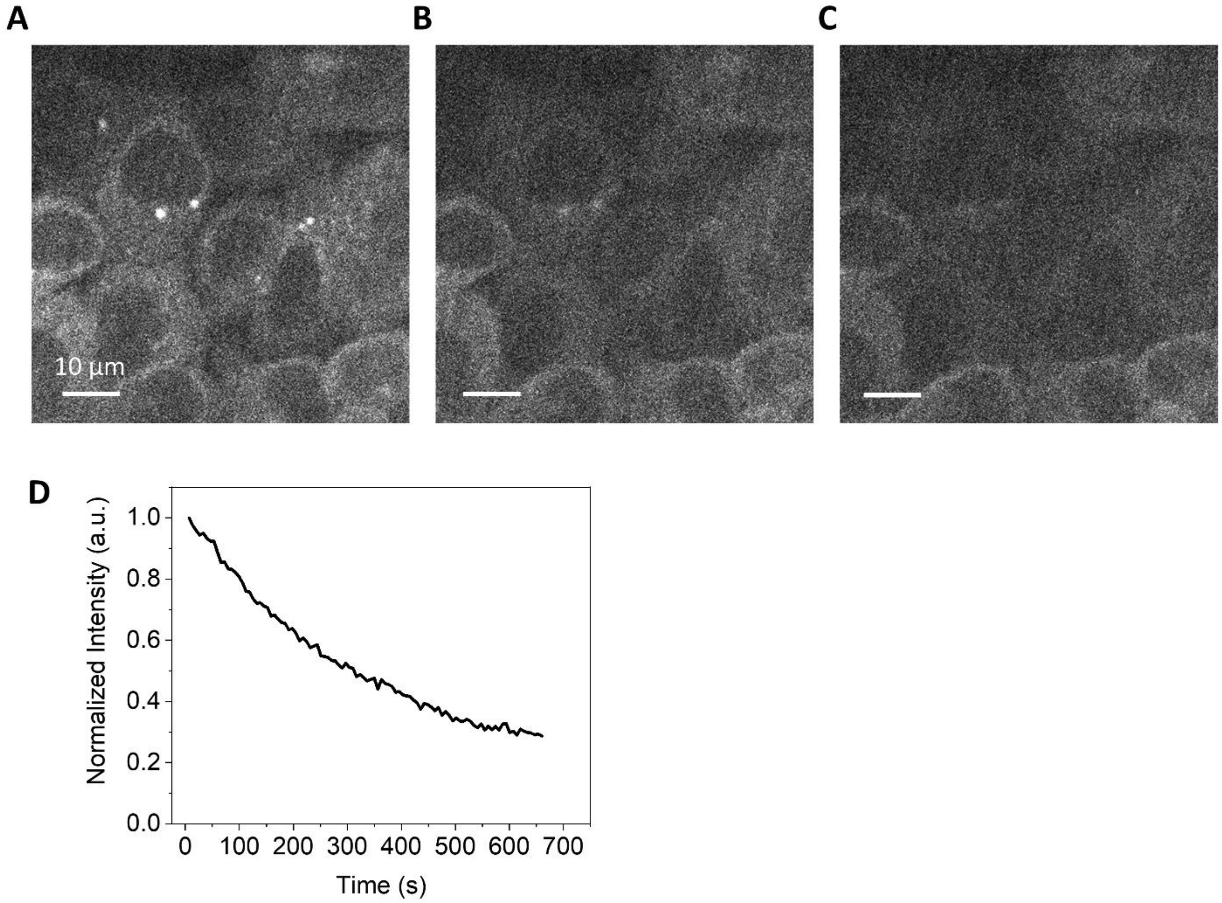

Figure S6. A–C) Time-lapse two-photon excitation fluorescence images of EB3-EGFP signals in HeLa cells acquired at different time intervals ((A): 0–6.6 s; (B): 330–336.6 s; (C): 660–666.6 s), using 10 mW 800 nm and 20 mW 1045 nm fs laser pulses (250 fs, 80 MHz) with a pixel dwell time of 30 µs. No clear EB3 comets, as observed in Figure S5, are detected. D) The EB3-EGFP signal changes as a function of imaging time. The fs excitation lasers exhibit significant photobleaching of EB3-EGFP signals.

Supporting Videos

Video S1. Real-time APX and MitoTracker signal changes for cells displayed in Figures 3B, C.

Video S2. Time-lapse EB3-EGFP signals for HeLa cells in Figure 3F.

Video S3. Time-lapse EB3-EGFP signals for HeLa cells in Figure 4B.

Video S4. Time-lapse EB3-EGFP signals for HeLa cells in Figure 4C.

Video S5. Time-lapse EB3-EGFP signals for HeLa cells in Figure S5.

Video S6. Time-lapse H2DCFDA signals from MIA PaCa2 cells after localized fs laser treatment shown in Figure 5A.

Video S7. Time-lapse H2DCFDA signals (green) and MitoTracker signals (red) overlaid from MIA PaCa2 cells after localized fs laser treatment shown in Figure 5A.

Video S8. Time-lapse EB3-EGFP signals for HeLa cells in Figure 6A.
